# Self-Reported Mental Health Benefits and Impacts of Vocational Skills Training in a Low-Resource Setting: The Lived Experience of Young Women Residing in the Urban Slums of Kampala, Uganda

**DOI:** 10.3390/ijerph22111698

**Published:** 2025-11-11

**Authors:** Monica H. Swahn, Matthew J. Lyons, Jennifer A. Wade-Berg, Jane Palmier, Anna Nabulya, Rogers Kasirye

**Affiliations:** 1 School of Public Health, Virginia Commonwealth University, Richmond, VA 23219, USA; 2 Department of Health Promotion and Physical Education, Wellstar College of Health and Human Services, Kennesaw State University, Kennesaw, GA 30144, USA; mlyons30@kennesaw.edu (M.J.L.); jwadeber@kennesaw.edu (J.A.W.-B.); palmierj@vcu.edu (J.P.); 3 Uganda Youth Development Link, Kampala P.O. Box 12659, Uganda; nkanna.kavuma@gmail.com (A.N.); kasiryer@yahoo.com (R.K.)

**Keywords:** vocational training, women, Uganda, empowerment, mental health, vulnerable population

## Abstract

Vocational training can lead to higher employment rates and improved incomes, particularly for young women in low-resource settings like Kampala’s slums. Despite these benefits, further research is needed to understand the full impact and mechanisms of vocational training on youth in low-resource environments. In 2022, a focus group project, part of a larger study, involved 60 women aged 18 to 24, recruited from three Youth Support Centers operated by the Uganda Youth Development Link (UYDEL) in Kampala. Six focus groups (about 10 women in each group) were held to explore urban stress and how vocational training might mitigate social and environmental stressors and improve mental health. Data analysis conducted using NVivo software identified five key themes: economic benefits, skill development, building confidence and self-esteem, improved social and behavioral well-being, and enhanced lifestyle and quality of life. This formative research underscores that vocational training benefits young women, highlighting outcomes such as job acquisition, financial empowerment, and skill development. Additionally, self-esteem and confidence development emphasize the training’s role in fostering mental health and agency and addressing gender inequality. These findings underscore the value of vocational training in enhancing the mental health and overall well-being of young women and suggest areas for future research for how to best optimize and scale these programs in low-resource settings.

## 1. Introduction

Vocational skills training offers numerous benefits for young people, particularly in terms of employment, economic empowerment, and overall well-being. These benefits are significant for young people living in low-resource settings [1]. Vocational skills training is an approach that directly contributes to the achievement of the Global Sustainable Development Goals, specifically Goal 1: End Poverty in all its forms; and Goal 8: To Promote inclusive and sustainable economic growth, employment, and decent work for all [2]. Vocational training is also aligned with the Uganda National Development Plan III (2020/21–2024/24), in which it is prioritized to enhance skills and vocational development and to address unemployment, especially among youth [3]. However, research on the benefits of vocational skills training is fragmented and lagging the growing scope of its implementation across countries and settings. Vocational skills training, also referred to as vocational education, encompasses a range of educational programs designed to equip individuals with specialized skills and knowledge tailored to specific jobs or trades. In contrast to traditional academic education, which prioritizes theory and general knowledge, vocational training typically focuses on practical, hands-on learning, preparing individuals for distinct careers or industries. The primary goal of vocational training is to provide individuals with the technical skills, competencies, and qualifications essential for employment in a particular occupation or industry.

While the research is sparse on the outcomes and benefits of vocational training, there is a particular dearth of studies in low-resource settings. As intended, vocational training has been associated with higher employment probabilities for women and increased wages for men, highlighting its positive impact on a range of positive and gender-specific outcomes in industrialized countries [4]. Moreover, vocational training during the transitional young adult period positively impacts employment status, job skills participation, and perceived preparedness for employment among young people in the U.S. [5]. Studies conducted in low-resource settings also indicate a range of potential benefits. For example, research has shown that vocational training plays a vital role in improving the economic status of youth in India, leading to increased income and expenditures on food, education, and health [6], and to improvement in family income and the ability to provide basic needs and shelter among young women in Uganda [7].

Similarly, vocational education is a beneficial option for motivating youth, providing them with the necessary skills for future success in the labor market [8]. In fact, vocational training enhances a wide range of life skills such as communication, motivation, teamwork, and responsibility, contributing to the overall capability of young individuals [9]. Vocational training programs are also identified to address youth unemployment, focusing on filling the gap between skills training and employment outcomes [10,11]. Additionally, vocational training has been associated with improved school success among students from low socio-economic backgrounds, highlighting its potential to promote educational attainment and social mobility [12].

Vocational training may also promote equity since it has been linked to improved wages and economic empowerment, particularly for ethnic minorities, demonstrating its potential to address disparities and promote financial stability among marginalized groups [13]. It is therefore likely a promising strategy for promoting equity among young women.

Vocational training may also have potential multifaceted health benefits, which have been studied across a few different contexts. Research shows that equipping individuals with vocational skills facilitates their contribution to economic development and improves their health and well-being [14]. Vocational training also plays a vital role in improving the economic status of youth, increasing their income, and enhancing their food, education, and health expenditures [6], and also as part of providing skills training in a trade such as tailoring and hairdressing [15]. Intriguingly, research also suggests that vocational training can contribute to the recovery of young people with mental illness, emphasizing the importance of integrating evidence-based vocational practices with quality mental health care to restore hope and inclusion [16,17]. This area of research warrants further examination given its promising findings and applications in settings where mental health resources are scarce. However, as emerging research continues to demonstrate the potential health benefits of vocational training, particularly in low-resource settings, it is essential to note that additional research is needed to evaluate the benefits and potential impact of vocational training, particularly for vulnerable youth in resource-poor settings with limited access to formal education [18].

Research on the potential positive benefits of vocational training remains fragmented across settings and countries, and definitions of both training and impact also vary significantly. However, vocational training offers potential benefits across a range of financial and health outcomes, particularly in low-resource settings. There are also intriguing findings about vocational training impacting equity, a particular concern among young women residing in slums who often feel marginalized and excluded from decision-making and who feel like they have less agency and control in their environment [19].

In the context of limited employment opportunities for out-of-school youth [20], challenges in highly populated urban job markets [21], and poor preparation for the current labor market, many students, including those with formal education, end up unemployed [22]. Moreover, vocational skills training has been identified as a significant determinant of access to the job market for marginalized slum youths, indicating its potential to create employment opportunities and improve livelihoods in slum communities [23]. Vocational skills training emerges as a potential solution that can offer alternative pathways for young individuals to acquire relevant skills and break the cycle of poverty [22].

The impact of poverty on access to formal education and student retention rates is evident, particularly affecting youth in slum areas [24]. Financial constraints, such as the cost of uniforms and supplies, contribute to many young people not enrolling in or failing to complete primary or secondary school [24]. This lack of access to formal education further widens disparities and exacerbates risk, as evidenced by research indicating lower condom use and other health-risk behaviors among out-of-school youth in slums compared to their urban, school-attending counterparts in Uganda [25].

Vocational skills training has the potential to impact the lives of people living in slums significantly. The literature suggests that vocational training can contribute to economic empowerment, gender equality, and mental health support for individuals in slum communities. Moreover, vocational training plays a crucial role in improving the financial status of individuals, leading to increased income and expenditures on essential needs such as food, education, and health. Additionally, it has been noted that vocational training can help break traditional barriers to female labor force participation, thereby promoting gender equality and economic independence [26]. Furthermore, vocational training has been associated with improved wages for ethnic minorities, highlighting its potential to address disparities and promote financial stability among marginalized groups [13]. Despite its potential, the scientific literature lacks comprehensive research and evaluations on the impact of vocational skills training, particularly concerning health behavior and mental health outcomes among youth in sub-Saharan African slums.

Urban slums are experiencing rapid growth, primarily driven by population expansion and rural-to-urban migration. Recent data from the UN-Habitat indicates that most urbanization will occur in low-income countries [27]. This accelerated urban growth may be of concern, particularly in sub-Saharan Africa, where more than 60% of the urban population resides in slums [28]. Uganda, identified as one of the countries with the highest population growth [29,30], has over half of its urban population estimated to be living in slums [31].

As such, slums hold significant importance as distinct places characterized by hardship. More specifically, the UN-HABITAT defines a slum household as a group of individuals living under the same roof in an urban area who lack one or more of the following: 1. Durable housing of a permanent nature that protects against extreme climate conditions. 2. Sufficient living space, which means not more than three people sharing the same room. 3. Easy access to safe water in sufficient amounts at an affordable price. 4. Access to adequate sanitation in the form of a private or public toilet shared by a reasonable number of people. 5. Security of tenure that prevents forced evictions [32,33]. Another term, “informal human settlements,” is frequently used to characterize slums, signifying locations with low-quality housing, overcrowding, pollution, and limited basic infrastructure, all contributing to unsafe residential conditions [27]. This terminology reinforces the interconnectedness between slums or informal human settlements and the health and welfare of young women and their view of place [18].

It is also important to consider the broader societal impact of vocational training in slum areas and its potential to uplift young women. Vocational education and training have been recognized as crucial factors in promoting self-employment, entrepreneurship development, and occupational mobility among young people, which can contribute to overall community development and economic growth [34]. As such, investing in young women and their vocational training may be particularly beneficial in low-resource settings and produce other positive benefits for the women and their communities.

It is clear that vocational training has the potential to bring about positive changes in the lives of individuals living in slums, including economic empowerment, gender equality, mental health support, and community development. By providing access to skills training, vocational education can contribute to breaking the cycle of poverty and creating pathways to a better future for individuals in slum communities. This may be particularly relevant in Uganda, where 50% of the population is 15 years or younger [30] and only 24% of adolescents are enrolled in secondary schools [35]. Intriguingly, Uganda is consistently ranked as one of the countries with the highest percentage of entrepreneurs (28%) [36], albeit primarily micro or small businesses. However, business failures are high, as youth lack basic budgeting and skills training in addition to other barriers to success. As such, vocational training, particularly for women, may be a strategic approach for financially uplifting women and supporting entrepreneurship.

While research demonstrates positive benefits from vocational training, research on the specific mechanism by which vocational training may lead to positive outcomes, from the trainees’ perspectives, remains lacking. In fact, few studies have sought to explain the key themes and context for how young women may feel empowered due to vocational training. The purpose of this formative qualitative study was to explore how vocational training influences empowerment, well-being, and livelihood opportunities among young women living in Kampala’s informal settlements. As such, we sought to understand how and through which pathways vocational training may improve women’s lives, including their psychosocial well-being and perceived agency. We could find no other studies to date to examine this issue among young women in similar settings. To our knowledge, no prior studies have examined this issue among young women in similar low-resource urban contexts. Given the urgent need for targeted interventions to promote health in these vulnerable populations, this is a critical area for intervention development and for informing policies that integrate economic and mental health promotion approaches.

## 2. Methods

The focus group project was conducted with 60 women, 18 to 24 years of age, in October 2022. Participants were recruited from three Youth Support Centers operated by the Uganda Youth Development Link (UYDEL) across metropolitan Kampala, Uganda. Participants were formerly enrolled in a vocational training program offered by the local community-based organization.

We conducted six focus groups (about 10 women in each group), two from each of the three study sites in Banda, Bwaise, and Makindye, to ensure each study site was adequately represented. The overarching goal of these focus groups was to understand “urban stress” and determine how the young women perceive their social and environmental stressors and their role in mental health outcomes. The guiding questions and prompts are presented in Table 1. The purpose of the analyses for this report was to understand and summarize the responses to the focus group prompts about how the young women perceive the vocational training they received and whether it helped mitigate their social and environmental stressors or mental health issues. This formative research was part of the larger TOPOWA study, designed to understand the impact of vocational and psychosocial skills training on young women’s mental health outcomes. Findings from the focus groups were incorporated into the design of a prospective, longitudinal study of young women.

### 2.1. Participants

The 60 participants were invited on a first-come, first-served basis to take part in the study. All potential participants were informed about the study and provided written informed consent prior to participating in the study. Ethical approvals were obtained from all relevant institutions. Participants were selected from the community-based organization’s drop-in centers for vulnerable youth ages 10–24 to provide psychosocial support, social services, and vocational skills training to vulnerable youth. The skills training provided includes cosmetology, catering, bakery, jewelry, tailoring, mechanical skills, and electrical skills, among others.

### 2.2. Procedure

The focus group discussions were led by two experienced women lead facilitators who were CITI-certified and had prior experience and training using this research approach. English is the official language in Uganda. However, the facilitators were fluent in both English and Luganda (the local language) and could facilitate translation as needed for participants. Focus groups were mostly conducted in Luganda and then translated into English. All the discussions commenced with the introduction of the moderators and participants of the study, along with descriptions of the context of the study. After the opening questions, the discussions were shifted to key questions and predetermined prompts to collect the required data. The women participants resided in one of three slums and were between the ages of 18 and 24 years of age. All women selected a pseudonym for the focus group discussion to ensure privacy.

### 2.3. Analysis Strategy

The data from the six focus groups were analyzed using NVivo 14 software. All of the focus group transcribed data was imported into NVivo to identify codes and themes.

Thematic analysis followed Braun and Clarke’s six-phase framework. Coding was conducted collaboratively by members of the research team, including NGO leadership from the Uganda Youth Development Link, who have extensive experience working with the target population. Initially, transcripts were read line by line to identify initial codes, and then initial codes were merged into themes [37]. The team met regularly to review transcripts, discuss emerging codes, and refine themes through discussion and consensus-building. Consistency was achieved through a collaborative and reflexive process involving iterative team discussion and joint review. We determined that data saturation was achieved when no new themes or perspectives emerged across the final sessions, and thematic patterns were consistently repeated across groups. Our analysis of the respondents’ views of vocational training comprises five themes, including (1) economic benefits, (2) skill development, (3) building confidence and self-esteem, (4) improved social and behavioral well-being, and (5) improved lifestyle and quality of life.

### 2.4. Reflexivity Statement

This study was conducted by a multidisciplinary and international research team whose diverse expertise and positionalities informed all stages of the research process. Dr. Monica H. Swahn, an epidemiologist and global health researcher with extensive experience conducting mixed-methods research in Uganda, served as Principal Investigator and provided overall conceptual and scientific leadership. Dr. Matthew Lyons, a mixed methods researcher and implementation scientist, led data analyses and interpretation, ensuring methodological rigor and consistency across themes. Drs. Jennifer A. Wade-Berg and Jane Palmier contributed expertise in community-engaged research, behavioral science, and implementation, supporting the contextual interpretation of findings. In Uganda, Ms. Anna Nabulya, Deputy Director of the Uganda Youth Development Link, coordinated participant recruitment, data collection, and focus group implementation, while Dr. Rogers Kasirye, Executive Director of the Uganda Youth Development Link, provided programmatic oversight and local contextual insight.

The team acknowledges several barriers inherent to international and community-based qualitative research, including logistical challenges, differences in disciplinary perspectives, and language translation that may have influenced data interpretation. Ongoing collaboration, frequent dialog across sites, and the leadership of Ugandan co-investigators helped mitigate these challenges. The combination of international and local perspectives allowed the team to balance insider and outsider viewpoints, ensure cultural and ethical sensitivity, and center the lived experiences of young women throughout the analytic and reporting process.

## 3. Results

### 3.1. Theme 1: Economic Benefits

The first theme, “Economic Benefits,” centers on the positive economic effects of the training programs and the financial empowerment women experienced because of participation. For example, participants shared that vocational training helped them to secure employment, with one respondent (FGD Makindye Group 2) stating, “I thank UYDEL, because when you graduate, they help you ‘bambi’, and find you a job. Now me I was at home and didn’t know what to do, I was confused, and then I saw a call from UYDEL asking if am working and they got me a job. Now it is where I am working so I really thank UYDEL.” Participants stated that vocational training was also supportive of healthy parenting and provided financial resources necessary for childbirth and child rearing. Furthermore, participants shared that the training also helped to keep them active and to learn employable skills that they can keep for the rest of their lives. Moreover, participants mentioned that now it is easier for them to start businesses after being equipped with these new skills learned. As one respondent (FGD Banda Group 1) shared, “even if you don’t have the money to start something you want to do but through the skill I have learned and gained, am able to start up something for example, a customer can approach you and they ask you to plait after which you get paid.”

Participants also discussed growing the customer base of their businesses, as well as the improved focus they experienced as a result, since their interactions with customers helped distract them from daily challenges. Finally, participants noted that the program helped them to earn and save money. As one participant (FGD Bwaise Group 2) shared, “Personally as fortune, with the fact that some of us the young girls gave birth and are mothers, once one is able to learn a vocational skill like hair dressing, you can start by beautifying the people around you, once you do a great job in beautifying them, then you can get to earn some money, which you could use to pay for the children’s needs like milk, or even school fees.”

### 3.2. Theme 2: Skill Development

The second theme, “Skill Development,” focuses on the developmental benefits of the programs and the ways in which new skills contributed to success and well-being. Participants shared that vocational training helped in acquiring the skill of tailoring, for example, with one respondent (FGD Makindye Group 1) stating, “I study tailoring at UYDEL, me I study tailoring for sure… It has really helped me because now I can sew.” Participants also noted that they acquired skills with teaching others, which had positive ripple effects for the community since they could help their fellow students and make a living by teaching others. Several participants noted the benefits of the skills they developed, and one participant (FGD Makindye Group 1) stated, “I was able to study vocational skills and playing girls football. This helps me not to be stressed and not to think about challenges at home.” As noted in this example, participants mentioned vocational skill development also in addition to athletic skill development, as a benefit of program involvement.

Participants also noted the ways in which the program helped them build and express their creativity, acquire materials to create new clothing designs, and to use these skills to build greater respect in the community. As one respondent (FGD Banda Group 2) mentioned, “While I was in senior four, and almost sitting my exams, I got pregnant then everyone said that I wasted my parents’ money because I got pregnant. So, I got worried and yet I couldn’t abort the pregnancy, so I later gave birth but I didn’t know what to do next, because I didn’t sit exams meaning I didn’t have any education certificate, what was I going to do, and yet I didn’t have any single skill for which to start with. But when I heard about UYDEL, right now they keep calling to ask “how is Lillian doing? And they tell them that I already learned hair dressing and now they want to talk to me and yet they had abandoned me since I was nothing.” Moreover, it also helps in learning skills instead of sitting idle; vocational skills help because when one lacks a skill, they are idle. Another respondent (FGD Makindye Group 1) mentioned, “For me, like Liz, the tailoring has helped me not to be bored, or to spend time loitering with boys. The time I would spend standing with a boy on the street, I spend it cutting my paper. The next day I come back when I have mastered the previous day learning.” Overall, participants noted significant benefits of skill development, including a greater chance to express creativity, greater economic opportunity, and greater respect in the community.

### 3.3. Theme 3: Building Confidence and Self-Esteem

The third theme, “Building Confidence and Self Esteem,” highlights the ways in which vocational training helped participants to become more confident in their daily lives. Participants shared that vocational training helped boost morale, self-efficacy, and determination to achieve goals. As one respondent (FGD Banda Group 1) stated, “right now they have gained confidence whereby they can stand up and reach out to the community confidently.” Participants also mentioned that the improvements in confidence led to improved communication and advocacy capacity and decreased fear of self-expression. Participants noted significant improvements in their sense of self-worth, self-belief, and sense of belonging in society. One respondent (FGD Banda Group 1) stated, “The skills they have acquired have contributed to building their self-esteem and believing in oneself because right now some of them are not creative enough but they can sit for Directorate of Industrial Training examinations which makes them to equate to those that are educated through obtaining a government certificate and since they have the ideas in their heads they can work effectively outside there.”

Participants noted that the training also helped them to develop better self-understanding, a better sense of what they want, and a greater capacity to demonstrate competency in the eyes of the community. Participants noted that the trainings helped them to feel empowered, with one respondent (FGD Banda Group 2) stating, “Well for me, vocational skills have helped me get confident and be able to talk to people. Because before I used to be scared and in case someone abused me on the way, I would fear to pass through the same way again, as I would think and they would abuse me again. However, right now I am no longer scared as I have confidence, and in case what someone tells me is right, I changed, in case it is not beneficial, I ignore it.” Participants shared that the vocational training helped them to overcome obstacles that had previously felt insurmountable. As one respondent (FGD Banda Group 2) shared, “When I got pregnant at a young age, I thought that everything had come to an end. But when I was told that UYDEL could help me, because my mother and father had abandoned me, literally everything had come to an end. That is how I came to UYDEL for hairdressing, but I am even the one that is helping these other colleagues.”

### 3.4. Theme 4: Improved Social and Behavioral Well-Being

The fourth theme, “Improved Social and Behavioural Wellbeing”, focuses on improvements in behavioral health and social network makeup as a result of program involvement. Participants mentioned that vocational training and the job opportunities that followed helped them to get out of dangerous professions (i.e., commercial sex work). As one respondent (FGD Banda Group 2) stated, “Me as flower, when UYDEL had just started, there are girls whom are new that were prostitutes and so were always in bars but since they accomplished their training, they all started up saloons and are always working, I believe they already left their first jobs of sex work for they no longer have time for it.” Participants also shared that vocational training helped them build healthy routines and reduce negative behaviors to attract customers for their legal businesses. One respondent (FGD Banda Group 2) said, “it has helped me because these days I wake up at 6:00 a.m. with my alarm and I also get some customers, so it has helped me to avoid being lazy like I was before.”

Participants also noted that the training contributed to reduced community conflict, with one respondent (FGD Bwaise Group 2) stating, “Personally as Faith, it is so true that vocational skills training has helped many children, and it has helped to reduce on the conflicts in the communities where we come from.” Participants also noted that the training helped to increase positive social influences and manage stress, with a respondent (FGD Makindye Group 2) stating, “at UYDEL I have made good friends, that sometimes I leave home with work stress and mine as a person, but when you seat somewhere and a student comes and tells you Joy, you are ‘boring’ yourself. How is your baby. You know when your baby is happy you are also happy. You feel the stress being released; you leave when the stress is over. Sometimes I don’t want to go home. When I am happy, my son is also happy because then I will not torture my him because out of stress. That even when he makes a mistake you let it slide because you are happy from UYDEL because of the comfort from friends.”

Participants noted that the vocational training program helped them develop better relationships with their families and faith communities as well. One respondent (FGD Makindye Group 2) said, “Before coming to UYDEL I was hurting my mom’s ‘head’, there was no bar I didn’t go to, but my friend told me about the free vocational school and I already had interest in vocational skills but my mom didn’t have the money. I had groups of girls and we were always in bars and my mother had given up on me, so my friend brought me to UYDEL. But UYDEL taught me to pray, I used to engage in scripture union and pray and this gave me peace even now on Sundays I do not miss praying. My mother is now proud of me because now I do not hang out in groups, I spend my time working I no longer have that time.” Overall, participants noted that the programs supported their behavioral health and also improved their quality of connection with others.

### 3.5. Theme 5: Improved Lifestyle and Quality of Life

The fifth theme, “Improved Lifestyle and Quality of Life,” focuses on improvements in lifestyle and quality of life. Participants overall reported that their quality of life was improved because of the training. As one participant (FGD Makindye Group 1) stated, “Me as Rovin, ‘entering’ UYDEL has helped me to change my life.” Participants noted that they had a greater sense of and capacity for responsibility after the training as well, with one participant (FGD Makindye Group 1) saying, “I am Sarah. Another thing, what I am grateful for at UYDEL, it has enabled me to become a responsible person.”

Participants mentioned that, after the training, they were able to become ambassadors for other youth and that the skills they developed were useful in sustaining a better quality of life. In addition, participants mentioned that the training helped them to deal with a variety of challenges that come up in life. As one respondent (FGD Makindye Group 1) shared, “I have experienced UYDEL, it has helped me to know how to go through any situation no matter what it looks like.” Furthermore, participants shared that it also helped in restoring their sense of hope for a better life, as well as their standing in the community. As one respondent (FGD Banda Group 1) shared, “The vocational skills we have acquired from UYDEL, have restored hope in us specifically the teenage mothers in the community are looked at as a symbol shame in the community and are demoralized.” Overall, participants reported that the numerous benefits of the training, including economic, social, educational, and psychological benefits, resulted in an overall improvement in their quality of life.

## 4. Discussion

The purpose of this study was to explore how vocational training influences empowerment, well-being, and livelihood opportunities among young women living in Kampala’s informal settlements. While prior research has documented the benefits of vocational training [4,6,11,13], few studies have examined how these programs create meaningful change from the participants’ own perspectives, particularly in low-resource African contexts [10,15,23]. In this formative research and qualitative assessment of the benefits of vocational training, which is likely the first of its kind, we gained great insight into how women perceive the training and how the training is most impactful to them in terms of mental health and well-being.

Through the analyses of the focus group data, we identified five key themes: (1) economic benefits, (2) skill development, (3) building confidence and self-esteem, (4) improved social and behavioral well-being, and (5) improved lifestyle and quality of life.

Some of these themes overlap, which is expected in qualitative thematic analysis, where interconnections among domains often reveal the depth and complexity of lived experiences. The interconnectedness across themes, particularly between economic empowerment, social well-being, and overall quality of life, reflects the interdependent and cumulative nature of change, whereby progress in one domain reinforces gains in others rather than existing as distinct or isolated outcomes. This overlap is conceptually meaningful, illustrating that participants’ experiences of empowerment and improved well-being are multidimensional and mutually reinforcing rather than having a linear causal structure. Such overlap is also consistent with qualitative and inductive approaches like thematic analysis, where interrelated themes often capture the complex and overlapping realities of participants’ lived experiences rather than neatly separated constructs.

The findings validated the expected economic and functional benefits of vocational training. These include helping women secure employment, achieve financial empowerment, earn a living, raise a family, and gain skills directly related to the intent of providing vocational training and specific skills to increase employability [1,5,34]. These results support the value of vocational training to uplift young women living in poverty in urban slums [7,15,23]. Furthermore, the results raise questions about how we can scale up vocational training to reach more women [8,11].

Other themes extended beyond economic outcomes, highlighting improvement in self-esteem, confidence, and agency, dimensions critically linked to gender equality and empowerment [9,16,17,26]. Participants also described becoming more creative, innovative, and resilient, as well as being better able to solve problems and cope with daily challenges [21].

Overall, the young women shared numerous examples of how the vocational training helped them to deal with proximal environmental stressors. As an example, the narratives suggest that vocational training not only provided alternative livelihood opportunities but also fostered meaningful behavioral and social transformation as shared by participants [18,20]. As examples, participants described leaving sex work, adopting healthier routines, and forming more positive social networks, indicating that the program may function as both an economic empowerment and behavioral health intervention that strengthens social connectedness and community well-being [15,18]. However, based on this qualitative approach, we cannot rigorously determine if the positive perceived outcome of vocational training on stressors reflected a direct effect or if the experience served to buffer stressors [15]. The young women reflected on improving social relationships and connections with family and their faith-based institutions, which may indirectly be related to reduced stress and empowerment [17]. However, this is an intriguing finding that can be posed as a research question to be addressed in future evaluations of the impact of vocational training in this or similar populations of young women. Additionally, while these findings contribute valuable insights into the psychosocial and empowerment benefits of vocational training, prior research exploring such outcomes among young women in similar low-resource settings is limited [10,23]. This gap makes direct comparisons with existing studies challenging but underscores the novelty and importance of this work.

Building on these novel insights, the findings further elucidate the potential mechanisms through which vocational training may shape both psychosocial and economic well-being among young women living in Kampala’s informal settlements. The results, while not constituting definitive evidence for causation, suggest several plausible interconnected pathways through which effects may take shape. Economic empowerment emerged as a foundational mechanism: the ability to earn an income and provide for one’s children reduced financial stress and fostered a sense of stability and control over daily life [1,6,15]. Skill acquisition, in their views, not only enhanced employability but also generated social recognition and self-worth, which helped participants regain dignity and overcome stigma associated with poverty, early pregnancy, or sex work [7,18,23]. Increased confidence and self-esteem appeared to translate into improved communication, problem-solving, and resilience in facing adversity [10,15,18]. Participation in vocational training also seemed to foster positive behavioral and social changes, including the development of structured daily routines, disengagement from high-risk environments, and strengthened peer and family relationships that may mitigate mental health risks. Finally, participants described a renewed sense of purpose and hope, suggesting that vocational training functions both as an economic and psychosocial intervention, promoting empowerment, reducing marginalization, and improving overall quality of life in low-resource urban settings [4,15,20].

There are some noteworthy limitations of this qualitative study. First, our participants were recruited from only three geographical areas in urban Kampala and also represented a narrow age range of women (18–24 years). This limitation was inherent, as the focus group discussions were part of formative research to inform a prospective cohort study of young women to assess the longer-term impact of vocational training on mental health and the social drivers in urban slums, as experienced by young women [38]. Second, we only included young women who had trained specifically at UYDEL. Many nonprofit and community-based organizations provide vocational training on various topics and with varying durations. As such, our findings may not represent other types of training or experiences. Third, as participants were self-selected, selection bias is possible, as women who chose to participate may have been more engaged or may have had more positive experiences with the program than non-participants. Moreover, because this is an exploratory qualitative study, the findings are not intended to be generalizable but rather to provide contextualized insights into participants’ lived experiences. Fourth, the focus groups were conducted primarily in Luganda and later translated for transcription and analysis in English. It is always possible that nuances and local references to context may not be picked up. However, a team from UYDEL and other Luganda speakers have reviewed the analyses and their interpretation to ensure the proper context. Fifth, given that participants were graduates of the UYDEL (the implementing NGO), the possibility of social desirability bias cannot be ruled out, as participants may have emphasized positive experiences or perceived benefits of the program. Also for context, this analysis specifically focused on the perceived benefits and positive impacts of vocational training; however, barriers and challenges experienced by participants are presented in a separate manuscript derived from the same study, which provides a complementary perspective on program limitations and contextual challenges. Sixth, as this was a formative qualitative study, triangulation with observational or quantitative data was beyond its scope but will be incorporated in future phases of the larger TOPOWA cohort study to enhance data credibility and analytic depth. Finally, while not a limitation, it is important for context to note that the UYDEL team did not conduct the thematic analyses of the focus group data in order to prevent any biases reflecting the benefits and impact on the vocational training they deliver.

Despite these limitations, the study offers several important insights for practice and policy. The findings have important policy and programmatic implications for NGOs, funders, and implementing partners working to advance gender equity, economic empowerment, and youth mental health in low-resource urban settings such as Kampala. In Uganda, where youth unemployment, gender-based violence, and limited access to mental health services remain pressing challenges, vocational training programs can serve as practical and sustainable entry points for addressing multiple development priorities simultaneously [1,3,10,15]. Strengthening such programs through sustained funding, mentorship, and integration with life skills and psychosocial support components could further enhance their long-term impact [5,6,15,17]. NGOs such as UYDEL are well-positioned to lead these approaches, drawing on their community trust and extensive experience in delivering youth-centered interventions [15,18,23]. Aligning these efforts with existing national priorities—such as those outlined in Uganda’s Third National Development Plan (NDP III), which emphasizes job creation, skills development, and social protection—can help ensure complementarity and scalability [3]. These priorities also align with global frameworks, including the Sustainable Development Goals (SDGs), particularly SDG 3 (Good Health and Well-Being), SDG 5 (Gender Equality), and SDG 8 (Decent Work and Economic Growth) [2]. For donors and international funders, these results highlight the value of supporting integrated initiatives that bridge economic empowerment, education, and health promotion, recognizing vocational training as a scalable strategy for improving livelihoods and well-being among young women in Uganda’s informal settlements [1,7,15,18].

## 5. Conclusions

This study adds important findings to the emerging literature that supports investment in vocational training for young women in urban slums and other low-resource settings [4]. The reflections from the young women in our study reinforce the notion that vocational training can help break the cycle of poverty and improve economic opportunities while also providing psychosocial benefits that reduce stress, enhance confidence, and strengthen social connections [16]. Theoretically, these findings contribute to a growing understanding of empowerment frameworks, illustrating how skill acquisition and financial independence can foster greater agency and self-efficacy among women facing structural disadvantage. Practically, the study highlights the importance of integrating vocational training with life skills, entrepreneurship, and mental health promotion components to maximize both economic and psychosocial outcomes. Socially, the findings underscore that, in the absence of many formal mental health promotion programs and interventions, vocational training may serve multiple purposes—supporting livelihoods, strengthening resilience, and enhancing social cohesion within communities in low-resource settings [18,25]. Future research should seek to disentangle the specific pathways by which such training programs yield positive outcomes and identify strategies to optimize delivery and scalability for young women in urban low-resource contexts.

## Figures and Tables

**Table 1:** Focus Group Questions and Prompts Used in the TOPOWA Project of Young Women in Kampala, Uganda.

Question	Associate Probes/Prompts
1. Tell us what you think about the vocational training you received at UYDEL.	a. Has it helped you? b. Have you felt more empowered?
2. What kind of stressors do you think young women who come to UYDEL face?	a. Family responsibilities b. Financial challenges c. Stigma d. Violence/abuse e. Alcohol or drug use f. Where they live g. Other stressors
3. Do you think the vocational training at UYDEL helps to address or reduce any of these stressors?	
4. How do women cope with feeling stressed, sad, or anxious?	a. Social support b. Alcohol or drug use c. Faith or church involvement d. Other coping strategies
5. What community factors do you think most impact women’s lives in the slums?	a. Access to latrines and hygiene b. Access to water spigots c. Sanitation d. Lack of health clinics or pharmacies e. Lack of food stores or kiosks f. Lack of privacy g. Crowding or room sharing h. Crime i. Violence j. Bars and dance clubs k. Pool, betting, or game halls l. Sex work and pleasure parlors m. Alcohol and drug use n. Alcohol marketing and promotion o. Other factors
6. Do you think that any of these community factors impact women’s stress and mental health (e.g., sadness, anxiety, alcohol or drug use)? Why or why not?	
7. Which of these community factors do you think are most important for women who may feel anxious, sad, or use alcohol or drugs?	
8. What environmental characteristics do your neighborhoods or communities have?	a. Trash mounds or collection areas b. Evidence of flooding c. Crumbling buildings d. Abandoned structures e. Open fires or trash burning f. Standing water or sewage g. Waterways, trenches, or gutters h. Electrical wires i. Barbed wire or fences j. Alcohol advertisements k. Communal water tap l. Latrines m. Livestock n. Vegetation or grass o. Clotheslines
9. Do these environmental characteristics impact women’s quality of life, stress, or mental health (e.g., sadness, anxiety, alcohol or drug use)? Why or why not?	

## Data Availability

The data used is presented in this qualitative report. However, upon reasonable request to the lead author, we can also share the original data files.

## Abbreviations

The following abbreviations are used in this manuscript:

FGD	Focus Group Discussion
UN	United Nations
UNICEF	United Nations Children’s Fund
UYDEL	Uganda Youth Development Link
